# Thyroid eye disease in the context of autoimmune polyglandular syndromes: comparative clinical insights from a retrospective study

**DOI:** 10.3389/fendo.2026.1824627

**Published:** 2026-07-24

**Authors:** Laura Croce, Elisa Gatta, Maria Gallo, Ilenia Pirola, Marsida Teliti, Francesca Coperchini, Flavia Magri, Carlo Cappelli, Mario Rotondi

**Affiliations:** 1 Department of Internal Medicine and Therapeutics, University of Pavia, Pavia, Italy; 2 Unit of Endocrinology and Metabolism, Laboratory for Endocrine Disruptors, Istituti Clinici Scientifici Maugeri Istituto di Ricovero e Cura a Carattere Scientifico (IRCCS), Pavia, Italy; 3 Department of Clinical and Experimental Sciences, Struttura Semplice Dipartimentale (SSD) Endocrinologia, University of Brescia, Azienda Socio Sanitaria Territoriale (ASST) Spedali Civili, Brescia, Italy; 4 Centro per la Diagnosi e Cura delle Neoplasie Endocrine e delle Malattie della Tiroide, University of Brescia, Brescia, Italy

**Keywords:** autoimmunity, Graves' disease, intravenous steroids, polyglandular autoimmune syndrome, thyroid eye disease

## Abstract

**Objectives:**

Thyroid Eye Disease (TED) may occur in patients with Graves’ disease (GD) either as an isolated condition (iGD) or in the context of autoimmune polyglandular syndromes (aGD). Whether the presence of associated autoimmune diseases influences the severity of TED remains controversial. This study aimed to evaluate the prevalence of autoimmune-associated Graves’ disease (aGD) among patients with moderate-to-severe, active TED requiring high-dose intravenous steroid therapy (IVS), and to characterize the associated autoimmune conditions.

**Methods:**

We conducted a retrospective multicenter study including GD patients treated with IVS for moderate-to-severe, active TED at two Italian tertiary referral centers. Patients were classified as having iGD or aGD based on the presence of concomitant autoimmune diseases. Demographic data, smoking status, thyroid parameters, steroid dose, and response to therapy were collected.

**Results:**

A total of 159 patients were included (mean age 51.7 ± 13.6 years; 27% males). Twelve patients (7.5%) had aGD, while 147 (92.5%) had iGD. Type 1 diabetes mellitus and rheumatoid arthritis were the most frequent associated autoimmune conditions. All aGD patients were female. No significant differences were observed between aGD and iGD patients in age, smoking status, cumulative steroid dose, or response to intravenous glucocorticoid therapy.

**Conclusions:**

Associated autoimmune diseases are uncommon among GD patients with moderate-to-severe TED requiring intravenous glucocorticoids. The possible role of poly-autoimmunity in the pathogenesis and progression of severe TED warrants confirmation in larger prospective studies.

## Introduction

Thyroid Eye Disease (TED), also known as Graves’ Orbitopathy (GO), is an inflammatory condition that involves the extraocular muscles and orbital fat (1, 2). The condition mostly occurs in patients with Graves’ hyperthyroidism, but some cases are diagnosed also in non-thyrotoxic patients with Graves’ disease (GD), or in euthyroid/hypothyroid patients without GD. The clinical prevalence of TED in the context of Graves’ disease is approximately 40% (3), although more recent series suggest a general decrease in the incidence of TED in the recent years (around 25%) (4). The clinical presentation of TED is mild in around 75% of cases, and it rarely threatens vision (1%) (5, 6). Established risk factors for TED include male sex (7), cigarette smoking (8), Type 2 Diabetes (9, 10), dyslipidemia (11), duration of hyperthyroidism (12), anti-TSHR antibodies (TRAb) titers (13) and, although debated, radioactive iodine (RAI) therapy (14). TED therapy is aimed at controlling inflammation, reducing symptoms, and improving quality of life. Mild forms can be managed through lifestyle modifications, control of thyroid function, selenium supplementation and local symptomatic measures (15, 16). Moderate-severe forms instead require, as first-line treatment, intravenous high-dose glucocorticoids (IVG) (e.g., methylprednisolone) (17, 18). While this therapy is able to improve the inflammatory response in 58-83% of patients, it has minimal effect on proptosis and variable results on diplopia. Moreover, relevant toxicity (mainly hepatic) can occur. Other therapies, such as mycophenolate, orbital radiotherapy (16) or Teprotumumab (a human monoclonal antibody that blocks the binding between IGF-1 and IGF-1R, thereby blocking the interaction with TSHR) (19) can also be employed. Also other immunosuppressive agents have been employed off-label, including Tofacitinib, Tocilizumab, Rituximab, Cyclosporine and Sirolimus (20, 21). Promising data have been obtained with statins, which may have beneficial effects in TED reducing cholesterol, inflammation, and fibrosis (22). TED can be complicated by sight-threatening complications, such as Thyroid Optic Neuropathy or corneal ulceration that may require surgical treatment (23).

GD can occur as an isolated condition or in the context of autoimmune polyglandular syndromes (24, 25). It is estimated that one third of GD patients present another autoimmune condition or will develop one during their lifetime. Nevertheless, the rate of associated autoimmune conditions varies across studies, ranging between 16.8% in the study by Ferrari et al (26), to 24% in the study by Gatta et al. (27) and between 8-21% in a meta-analysis by Botello et al (28). Few previous studies evaluated whether patients with isolated GD (iGD) and those with GD associated with other autoimmune conditions (aGD) show different prevalence of TED. In a small case-control study, Rotondi et al, reported similar rates and severity of TED between iGD and aGD patients (29), while Ferrari et al. found lower rates of TED in iGD patients compared with aGD (26). More recently, Gatta et al, in a large series of consecutive GD patients (27) reported higher prevalence and severity of TED in iGD patients as compared to aGD.

In order to reduce possible biases due to the lack of systematic evaluation of mild TED in GD patients, the present multicenter study was specifically designed for evaluating the proportion of iGD and aGD among GD patients with moderate-severe and active TED requiring high-dose iv steroid therapy (IVG). As a secondary objective, the specific type of associated autoimmune disease in the aGD cohort was also considered.

## Materials and methods

### Patients

The inpatients’ Data Base of the Unit of Endocrinology and Metabolism of two referral centers were retrospectively searched for patients receiving intravenous corticosteroids for TED in addition to standard therapy (i.e. Selenium supplementation and local measures such as lubricating eye drops). Inclusion criteria were: 1) availability of a detailed clinical history; 2) availability of a full thyroid work-up at disease diagnosis, including thyroid ultrasound and serum levels of TSH, FT4, FT3, thyroglobulin antibody (TgAb), thyroid peroxidase antibody (TPOAb), and TSH-receptor antibody (TRAb).

Patients were all affected by moderate/severe active TED as classified according to EUGOGO guidelines and stratified according to an iGD (isolated GD) and aGD (GD associated with other autoimmune disorders) diagnosis. All patients were of Caucasian ethnicity.

Graves’ disease was diagnosed in the presence of low serum TSH and high free thyroid hormones levels associated with positive TRAb tests. The ultrasound scan of the thyroid gland was consistent with a diagnosis of GD, including evidence of an increased thyroid blood flow. Thyroid volume was calculated with the ellipsoid formula.

Among the aGD group, several associated autoimmune diseases were reported and these were diagnosed according to current clinical guidelines. Associated autoimmune endocrine diseases were managed in our Endocrine Units. Non-endocrine autoimmune conditions were managed by the correspondent specialists. All patients signed the specific informed consent and data collected remained strictly confidential and anonymous, according to the ethical rules of our Hospital institutions and to the Declaration of Helsinki. The study was approved by the Ethical Committee “Comitato Etico Lombardia 6” (Protocol n ASST_BS_LifeSPA-NP 6674).

### Statistical analysis

Statistical analysis was performed using SPSS software (SPSS, Inc.,Evanston, IL). Statistical analysis was performed using SPSS software. Between-group comparisons were performed by means of Student t test for unpaired data and Mann–Whitney U test according to a normal or a nonparametric distribution of the variable tested. Frequencies among groups were compared by χ2 test with Fisher’s correction, when appropriate. A p value < 0.05 was considered statistically significant.

## Results

The study group included a total of 159 patients with TED who had undergone IVS at high doses (mean dose 4.9 ± 0.9 gr). The mean age was 51.7 ± 13.6 years. Forty-three patients were males (27%). Thirty-three patients (20.8%) were active smokers. The mean Clinical Activity Score (CAS) was 4.5 ± 1.2. Patients with iGD were 147 (92.5%) while only 12 patients (7.5%) had aGD. As shown in **Table 1**, among these 12 subjects the associated autoimmune diseases were Type 1 Diabetes Mellitus (T1DM) in 5 out of 12 cases (41.7%), Rheumatoid Arthritis in 3 out of 12 cases (25.0%), Vitiligo in 1 case (8.3%), Atrophic gastritis in 1 case (8.3%), Systemic Lupus Erythematosus 1 case (8.3%), Immune Thrombocytopenia (ITP) in 1 case (8.3%). One patient had both T1DM and Coeliac Disease. When compared with patients with iGD, patients with aGD had similar CAS scores (4.5 ± 1.2 in iGD vs 4.6 ± 1.1 in aGD, p=0.768), received similar doses of steroid therapy (4.9 ± 1.0 in aGD vs 4.9 ± 0.9 in iGD, p=0.921) and had similar age (50.9 ± 14.5 in aGD vs 51.76 ± 13.6 in iGD, p=0.838). All 12 aGD patients were females, while 29% of patients with iGD were males (p=0.028). No significant differences in terms of rate of active smokers (21.8% in iGD vs 8.3% in aGD, p=0.270), or of clinical response to IVS therapy (68.6% in iGD vs 80.0% in aGD, p=0.455) could be observed between the two groups.

**Table 1:** Autoimmune diseases associated with GD in the cohort of patients with TED receiving IV steroids.

Autoimmune conditions	n	%
Diabetes Mellitus type 1	4	33.3%
Rheumatoid Arthritis	3	25.0%
Vitiligo	1	8.0%
Atrophic gastritis	1	8.0%
Systemic Lupus Erythematosus	1	8.0%
Immune Thrombocytopenia	1	8.0%
Diabetes Mellitus type 1 + Coeliac Disease	1	8.0%

## Discussion

The results of the present study show that patients with moderate-severe GD-associated TED receiving IVS have a 7.5% rate of associated autoimmune conditions, being T1DM and RA the most frequently associated autoimmune conditions.

The 7.5% rate of aGD among patients with TED receiving IVS should be discussed in light of previous studies addressing the issue of iGD/aGD ratio in large consecutive series. Indeed, in a recent Italian study encompassing a large cohort of consecutive GD patient, stratified according to the presence of other autoimmune diseases (27), the Authors showed that patients with iGD developed TED more frequently than GD-APS patients and the severity of the ocular condition was significantly higher in patients with iGD. More specifically a 28% aGD/iGD ratio in the GRAPHE study was reported.

By comparison of the results provided by the present and the GRAPHE study, it should be highlighted that the two series were largely different for the aGD/iGD ratios (28% versus 7.5%). This significant difference allows expanding the finding of the GRAPHE study by stating that not only aGD would develop less frequently TED, but also that its severity would be lower, as demonstrated by a reduction in the aGD/iGD ratio when the comparison is limited to moderate severe cases requiring IVS.

A certain inconsistency in literature exists on this topic. Indeed, in a previous study by Ferrari et al., it was found that patients with TED had another associated autoimmune disease more frequently (18.9%) than patients with GD without TED (15.6%). No association with the type of associated autoimmune disease and the presence of TED was observed. Moreover, no evaluation of TED severity or degree of activity was performed (26). In another previous study by Rotondi et al, no differences in the rate and severity of TED was observed between iGD and aGD patients (29). These discrepancies could be in part explained by differences in study design (longitudinal cohorts vs case-control studies) and in the rates of aGD (17% in the study by Ferrari et al. vs 24% in the study by Gatta et al). Moreover, while the incidence of TED was similar in these studies (30% of patients in the study by Ferrari et al, 26% in the study by Rotondi et al. and in 29.5% in the study by Gatta et al), patients in the study by Gatta et al. displayed a more severe phenotype with only 43% of patients having a mild presentation of TED, when compared with the series by Rotondi et al, in which 60% of patients had a mild presentation. Lastly, it should be acknowledged that previous studies differ also in terms of prevalence of autoimmune comorbidities. Indeed, the most prevalent autoimmune comorbidities were Vitiligo and Autoimmune Gastritis in the study by Ferrari et al. and Rotondi et al, while T1DM and Coeliac Disease were the most prevalent in the one by Gatta et al. These discrepancies may be due to differences in local screening procedures and may have impacted the final result.

The results of the present study do not allow to draw firm conclusions regarding the reason underlying this phenomenon. Nevertheless, some hypotheses can be made.

As concerns the issue of previous immunosuppressive therapy in subjects with orbitopathy, in our case only one patient undergoing intravenous steroid bolus therapy had previously taken steroid therapy for rheumatoid arthritis.

This association has been previously studied by Kelada’s group, which found a lower degree of TED severity in patients with APS who had previously undergone immunosuppressive therapy compared to patients with poly-autoimmunity who had not received such therapy (30). Nevertheless, it should be highlighted that the study by Gatta et al. showed a lower rate of TED in aGD patients even after removing all patients who had received immunosuppressive therapies.

Another possible explanation could rely on the possible role of non-thyroid related autoantibodies in patients with TED who also have other autoimmune diseases. Indeed, a recent study by Lanzolla et al. showed that, although the rate of anti-nuclear antibodies was similar between GD patients with or without TED, their presence was associated with a less-severe TED phenotype (31). Nevertheless, the presence of non-thyroid autoantibodies was not systematically evaluated in the patients included in the present study.

As a further point, the aGD group included a higher percentage of women when compared with the iGD group. This finding is probably related with the generally higher prevalence of polyautoimmunity among women. Since male gender is associated with a worse TED presentation and response to therapy (32), we cannot exclude that this small difference may have played as a confounder in the comparison of the clinical response to IVs.

Potential limitations of the present study are its retrospective nature and the limited number of included subjects, especially those of aGD. The low number of aGD subjects may have limited, in the present study, the ability to discern patterns of association with specific subtypes of associated autoimmune diseases. Nevertheless, the inclusion of a specifically selected, even if rare, population, (i.e. subjects with a particularly severe presentation of TED requiring IVS) increases the strength of our finding despite the low numerosity. Moreover, since the diagnosis of other autoimmune diseases was performed based on clinical presentation, we cannot exclude that a limited number of iGD patients may have harbored asymptomatic autoimmune conditions, even if the number of involved patients is presumably low.

In conclusion, the results of the present study show that among GD patients with moderate-severe TED requiring IVS a low prevalence of other autoimmune conditions is observed. However, given relevant discrepancies with previous literature, the hypothesis that poly-autoimmunity may be protective towards the development of severe TED will need further confirmation in larger, multicenter, prospective studies encompassing patients harboring the whole spectrum of severity of TED.

## Data Availability

The raw data supporting the conclusions of this article will be made available by the authors, without undue reservation.
